# Integrated Assessment of Productive, Environmental, and Social Performances of Adopting Low-Protein Diets Technology for Laying Hens

**DOI:** 10.3390/ani15020146

**Published:** 2025-01-09

**Authors:** Dongsheng Li, Xiaoying Zhang, Zhiyang Zhao, Siqi Wang, Jing Wang, Hongliang Wang

**Affiliations:** 1 State Key Laboratory of Nutrient Use and Management, College of Resources and Environmental Sciences, China Agricultural University, Beijing 100193, China; lidongsheng@cau.edu.cn (D.L.);; 2 Key Laboratory of Feed Biotechnology of Ministry of Agriculture & Rural Affairs, Institute of Feed Research, Chinese Academy of Agricultural Sciences, Beijing 100081, China

**Keywords:** laying hens, life-cycle assessment, meta-analysis, N footprint, low-protein diet

## Abstract

China, the world’s largest egg producer, faces challenges of protein feed shortages and nitrogen pollution from manure. Low-protein diets (LPDs) offer potential solutions, but their adoption by small-scale farmers is uncertain. This study used surveys, meta-analysis, life-cycle assessment, and life-cost analysis to address these gaps. A field survey in Quzhou County (a typical representative of small- and medium-scale farmers in China’s egg farming industry) found that 84% of egg farmers were unfamiliar with LPDs and believed higher protein levels were better. On average, pre-laying and late-laying diets exceeded national LPD recommendations by 0.3% and 0.5% crude protein (CP), respectively. The main barriers to LPD adoption were perceived low quality, lack of veterinary recommendations, and limited technical support. A meta-analysis of 30 studies (published before July 2024) showed that reducing CP by less than 1% did not compromise productivity. Local LPD demonstrations and training improved farmer confidence. Results indicated a 10% reduction in the nitrogen footprint and a 115% increase in earnings for LPD-demonstration farmers. The study suggests a coupled pathway integrating research and technology diffusion, highlighting the need for collaboration among scientists, feed companies, and technical experts to overcome misconceptions and promote LPD adoption. This integrated framework offers valuable insights for sustainable agricultural development in China and other resource-limited countries.

## Simple Summary

Small-scale egg farmers in China face challenges related to a shortage of protein feed and environmental pollution caused by excessive nitrogen in manure. To address these problems, a sustainable feeding solution known as low-protein diets (LPDs) has been proposed. However, many farmers are unaware of this option, and some believe that more protein in feed is always better for egg production. This study aimed to understand farmers’ awareness and attitudes toward LPDs and to evaluate its environmental and economic benefits. By surveying farmers in Quzhou County, the study found that most had never heard of LPDs, and that those who did typically did not use it. The study also used methods such as life-cycle assessment and cost analysis to show that adopting LPDs can reduce nitrogen pollution and lower feed costs. The findings suggest that more education and support are needed to encourage the use of LPDs, which could lead to more sustainable farming practices and better environmental outcomes. This research provides important insights into how science can help improve agricultural practices and support environmental protection.

## 1. Introduction

Global livestock production is continuously increasing, particularly in emerging economies, driven by population growth and rising consumption of animal-sourced food as wealth improves [1]. Concomitant with the growth of animal production, increased quantities of feed ingredients are consumed. China has a shortage of feed resources, especially for protein feeds. As the largest soybean importer in the world since 2002 [2,3], China imported 36.57 million tons of feed protein in 2022 (https://data.stats.gov.cn, accessed on 19 October 2024). Moreover, projections indicate that China’s feed protein deficit will soar to 60.5 million tons by 2030 [4]. This shortage of feed resources, particularly protein feeds, poses a significant challenge to the sustainable development of livestock farming in China.

Simultaneously, nitrogen (N) losses from livestock production are exerting immense pressure on the environment. Research shows that 60–70% of the N in feed (i.e., mainly supplied by protein ingredients) is excreted by feces and urine, significantly contributing to ammonia (NH_3_) and nitrous oxide (N_2_O) emissions, which exacerbate air and water pollution [5,6]. Livestock production accounts for around 40% of the global anthropogenic NH_3_ and N_2_O emissions [7,8]. By 2050, about 76% of the total N entering the crop–livestock system is lost [9]. Thus, identifying feeding strategies that balance production performance with environmental sustainability has become increasingly urgent. It is well known that there is a strong correlation between dietary CP content and excreta N emissions [10]. Precision-feeding technologies, especially for LPD technology, have been found effective in N excretion reduction [11,12]. This technology is based on the essence of protein nutrition and the theory of amino acid nutritional balance to lower the feed CP content by supplementing the appropriate types and quantities of synthetic amino acids to accurately meet the nutritional needs of breeding animals. Thus, the LPD technology will not affect the performance of animal production and product quality. Application of LPD technology can also greatly save feed protein resources. It is estimated that every 1% reduction in dietary CP content can reduce soybean meal usage in feed formula by 2% [13], equivalently saving 10 million tons of soybean meal or 14 million tons of soybean per year in China.

Since the 1980s, China has consistently maintained its leading position in global egg production. In 2022, China’s egg production reached 29.38 million tons, accounting for 34.6% of global egg production (FAOSTAT, https://www.fao.org/, accessed on 19 October 2024). Approximately 56.9% of hens in China are raised on small-scale farms, each accommodating fewer than 10,000 hens. The primary reasons for this are limited capital investment for expanding farming scale, restricted availability of land, and constrained management skills [14]. To save costs, small-scale egg farmers tend to purchase pre-mixed ingredients to produce complete feed [15]. However, due to misconceptions, farmers often excessively increase the proportion of soybean meal in the recommended formula to prevent degradation of productivity and alleviate various stresses, such as high temperatures and diseases [16].

The 2021 “Technical Scheme for Reducing Corn and Soybean Meal in Pig and Chicken Feed” (https://www.moa.gov.cn, accessed on 19 October 2024) promoted LPD-adoption, but uptake remains low among small-and medium-scale farms [1,16]. Understanding stakeholders’ perceptions is key to improving adoption and shaping national promotion strategies [17,18]. Undoubtedly, the prerequisite for farmers to accept advanced agricultural technology is effectual and cost-effective. Recent studies on the effects of LPDs on egg production and egg quality have yielded mixed results. For instance, several studies [19,20,21] demonstrated that reducing dietary CP by 2.0–2.4% did not adversely affect laying hens’ productivity, including egg production, egg weight, and feed-conversion efficiency, particularly when supplemented with essential amino acids. Conversely, other studies [22,23,24] reported that reductions in CP content ranging from 0.45% to 4.01% led to declines in body weight, egg production, and egg quality (e.g., shell strength and albumen height). These discrepancies likely arise from variations in experimental conditions, hen breeds, and amino acid-supplementation strategies. Thus, a thorough literature review and synthetic analysis is essential to ascertain the precise impacts of LPDs on egg production. LCA (Life-Cycle Assessment) and LCC (Life-Cycle Costing) analyses have been widely used for assessing environmental and economic cost-effectiveness of agricultural production from a full life-cycle perspective [25,26,27], but their application to poultry farming for assessing the benefits of LPD technology remains limited. Quantitative evaluations of the environmental and economic aspects of LPDS in small-scale farming contexts are critical to providing actionable insights for farmers, policymakers, and other stakeholders, ultimately fostering broader adoption of this technology.

**Figure 1:**
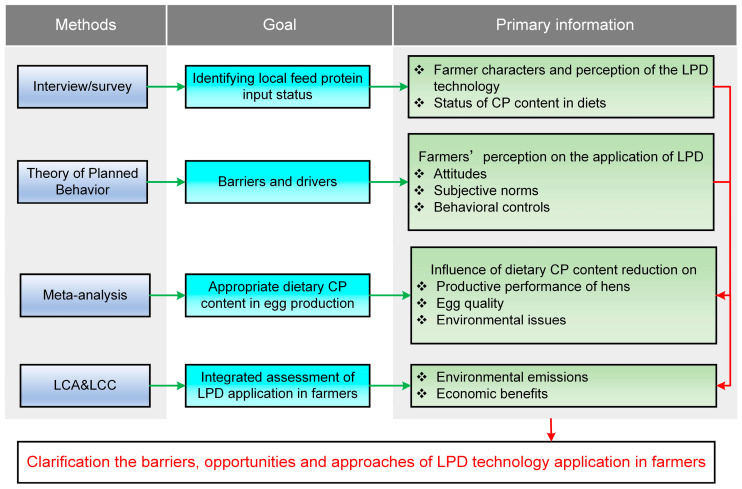
Schematic diagram showing framework of the study.

Hence, we firstly conducted a face-to-face farm interview based on the approach of Theory of Planned Behavior (TPB) [28,29] to capture the current production status of laying hen farmers in Quzhou County and to understand the driving forces and obstacles associated with the adoption of LPD technology among small-scale laying hen farmers. To further ascertain the extent of reduction in dietary CP content of laying hens, we conducted a meta-analysis of relevant studies and quantitatively analyzed the impact of LPDs on laying hen productivity. Lastly, we empirically assessed the environmental and economic benefits of egg farmers who adopted the LPD technology or not through LCA and LCC methods. The framework of this study is illustrated in Figure 1. The primary focus of this study, therefore, centers on understanding the drivers and barriers to LPD adoption among small-scale laying hen farmers in the North China Plain, along with the ensuing environmental and economic benefits. The aim is to provide policymakers, farmers, and stakeholders with a nuanced understanding of the feasibility and actual benefits of LPDs on egg production in China, thereby fostering the sustainable development of the poultry industry.

## 2. Materials and Methods

### 2.1. Theory of Planned Behavior (TPB) Methodology

A survey questionnaire was designed to conduct face-to-face farm interviews, structured based on the Theory of Planned Behavior (TPB) framework. Figure S1 illustrates the TPB framework, which explains that the intention to perform a certain behavior is influenced by three key elements: (1) attitudes toward the outcome of suggested changes, (2) subjective norms (social pressures to embrace suggested changes) and (3) perceived behavioral control (the perception of the ability to exert control over suggested changes). The TPB has been effectively employed in agricultural contexts to identify motivations and barriers to apply certain solutions [29,30,31,32].

#### 2.1.1. Questionnaire Design and Statistical Analysis

This study designed two questionnaires for the farm survey according to the TPB. Firstly, a semi-structured questionnaire was adopted to investigate how LPD technology is perceived by farmers, as well as to identify relevant outcomes, social referents, and controlling factors that influence LPD practices. A total of 15 layer farmers, three agricultural experts, and two officials of the local agricultural bureaus were involved in these semi-structured interviews in April 2021. In the interviews, participants were asked to identify: (i) the outcomes they would expect from the application of LPD technology, (ii) the referents who support LPD technology adoption or hinder it, and (iii) the factors that make applying LPD technology easy or difficult. Productivity factors were the most frequently stated dimensions such as body weight of spent hens, feed intake, feed efficiency, eggshell quality, heat stress, and gut health of hens. These factors can be either termed as barriers or drivers. Additionally, financial factors (e.g., saving feed cost, more labor needed), social factors (e.g., limits to acquiring information about LPDs, social referents), and environmental factors (reducing odor) were also mentioned. In total, 35 factors (12 outcomes, 15 social referents and eight control factors) were identified as influencing farmers’ use of LPDs. Based on this information, a second questionnaire was developed to obtain a more quantitative understanding of farmers’ perceptions of LPDs.

The outcomes, social referents, and controlling factors were quantifiably assessed. The scores of farmers’ perceptions regarding LPD technology were obtained through a detailed structured questionnaire. The questionnaire included six main sections: farm/farmer characteristics, farmers’ attitudes, subjective norms, perceived behavior control, farmers’ intentions to use LPDs with different drivers, and feed formula of layer farm. In addition, a Likert scale ranging from 1 to 5, with 5 representing very good or very well possible and 1 indicating very bad or not possible, was applied to score the farmers’ attitudes toward outcomes and belief strength, their perceptions of referents and motivations to comply, and their perceptions of controlling factors and control strength.

The below math equations were used to calculate the scores for outcomes (*i*), social referents (*j*), and controlling factors (*k*):

(1) $${attitude}_{i}={belief\;strength}_{i}\times \left({outcome\;valuation}_{i}-3\right)$$

(2) $${subjective\;norm}_{j}={motivation\;to\;comply}_{j}\times \left({normative\;belief}_{j}-3\right)$$

(3) $${behavior\;control}_{k}={control\;strength}_{k}\times \left({control\;power}_{k}-3\right)$$

The outcome scores were deliberately lowered by 3 points to acquire a balanced scaling range from −2 to +2. The attitude values ranged between −10 and +10. Furthermore, an attitude value greater than zero was termed a driver whereas the attitude value less than zero was termed a barrier. A belief strength lower than three indicates that the outcome’s probability is low. Hence, the outcomes with a belief strength lower than three are categorized as a potential barriers or drivers.

A one-way ANOVA was conducted to determine the statistical significance of differences in outcomes, social referents, control factors, and layer farmers’ intentions. Besides this, Spearman’s rank correlation test (SPSS 20.0) was used to analyse the correlation between the farmers’ intentions to use LPD and farmers’ and farms’ characteristics. At *p* < 0.05, differences were deemed statistically significant.

#### 2.1.2. Farm Survey and Feed Sampling

The study was conducted in Quzhou County, Hebei Province, China, which is a typical large agricultural county in the North China Plain, farming and stock raising are both the main economic origin of farmers. There were nearly ten million hens stocked in 2021, accounting for 49% of the gross agricultural product (Statistical yearbook of Quzhou County, 2022; http://tjj.qz.gov.cn, accessed on 19 October 2024). Layer farming in this county was predominantly based on conventional cage systems, with farms mostly being small-scale (less than 10,000 hens) or medium-sized (10,000–29,999), reflecting the majority of farm scales and types in the North China Plain [14] (Table S1). Table S1 provides detailed information on the scale distribution of layer farms, including the number and percentage of farms in each size category, as well as the cumulative percentage of hens housed. Four villages with the most stocked hens were chosen as the survey subject (Figure S2). This study interviewed a total of 100 farmers including 20 farmers who had received LPD technical training. However, four farmers failed to submit the questionnaire on farmers’ perceptions and thus were excluded. Finally, a total of 96 questionnaires were obtained and examined in this paper. Table S2 shows the basic characteristics of layer farmers. Subsequently, we engaged in further communication with the 20 farmers who had received initial training, including discussions with representatives from enterprises, the government, and the Science and Technology Backyard (STB). Based on these discussions, training content was designed and implemented to promote low-protein technology. Finally, 5 farmers who fully adopted low-protein technology were selected as demonstration households. Through these demonstration households, LCC and LCA methods were used to compare the environmental and economic differences between farmers who adopted low-protein technology (N = 5, LPD) and those who did not (N = 91, HPD).

Four well-trained master’s students from China Agricultural University performed this survey for a period ranging from early May to mid-June 2021. The training was extended to the enumerator students before the survey to introduce the survey objective, questionnaire, key concepts of LPDs, and the general situation of egg production. When they completed the questionnaire, the enumerator students would request a small amount of feed sampling (approximately 200 g) from the surveyed farmer. The great majority of farmers agreed with the request, and very few people refused us. Thus, a total of 94 feed samples were collected, excluding 2 samples from young hens. Small layer farms used similar feed ingredients (i.e., maize-soybean meal-based feed), namely maize, wheat bran, soybean meal, oils, stone powder and premix (Table S3). Feed samples were kept until analysis at −20 °C. The feed samples were firstly ground and passed through a 1 mm screen prior to chemical analysis. Afterward, these samples were analyzed for dry matter and CP (N × 6.25) according to the methods of the Association of Official Analytical Chemists (https://www.aoac.org/, accessed on 19 October 2024).

### 2.2. Meta-Analysis Methodology

To assess the effects of LPD technology on production performance, egg quality, and environment of laying hen, a meta-analysis based on peer-reviewed literatures published before July 2024 was conducted using the Web of Science and China National Knowledge Infrastructure database. Specific search terms were combined, related to dietary CP content (e.g., low protein diet, reduced CP, dietary protein, CP level), poultry (e.g., laying hens, layers, and hens), productive performance (e.g., egg production, egg quality, feed intake, feed efficiency, and feed–gain and gain–feed ratios). To be included in the systematic review, studies met the following criteria: (a) in-vivo laying hen studies including a control treatment group with normal dietary CP contents; (b) studies published in English or Chinese; (c) studies report productive performance [egg production (%), egg weight (g), egg mass (g/d/bird), feed intake (g), and feed–gain ratio or feed efficiency (FE), or gain–feed ratio (G:F) or feed–conversion ratio (FCR)]; (d) birds must have been free of diseases; (e) studies report sample variance (SD or SEM), sample size (n), and the age and duration of the study. All feed efficiency metrics recorded were converted to FE so that feed efficiency could be compared between experiments. Finally, 30 peer-reviewed studies were selected (Figure S3), and the amino acid balance was considered for diets in all studies. In this study, the response variables that were extracted for the meta-analysis included: egg production, egg weight, egg mass, and feed intake and feed efficiency of laying hens. Additional data were extracted, such as: features of the published study (author, year and place of publication), characteristics of the animals (sample size, age, and breed) and characteristics of treatments (dietary CP content and duration). The meta-analysis process was adopted from an early study of our authors [33].

Impacts of LPD technology on variables (*X*) were evaluated using the treatment against the pairwise control. The natural logarithm of response ratio (ln RR) can be calculated by the following equation [34]:

(4) $$\ln RR=\ln\frac{X_{t}}{X_{c}}$$

where ln *RR* refers to effect size for each paired observation, and *X_t_* and *X_c_* represent the average of the treatment and control, respectively. The weight of each observation was computed as:

(5) $$Weight=\frac{N_{t}\times N_{c}}{N_{t}+N_{c}}$$

where *N_t_* and *N_c_* are the number of repetitions of the treatment and control, respectively. The weighted response ratios and confidence intervals (CI, at a 95% level) were calculated by bootstrapping resampling procedures (4999 iterations) of R (version 4.0.2). The result was considered significantly different if 95% CI did not overlap zero.

### 2.3. Calculations of Nr Losses

Life-cycle footprint analysis was applied to quantify Nr losses of egg production. The functional unit is a carton of eggs (18 kg). The system boundaries extended from the cradle to the farm gate, divided into four components: (a) resource production, (b) feed production, (c) hens breeding, and (d) manure management (Figure S7). In this study, only chemical fertilizers and manure were considered as Nr sources (see details in Supplementary Materials).

### 2.4. Life-Cycle Costing and Profitability

According to the categories of LCC as detailed by [35], the conventional LCC generally includes costs that are associated with products or services borne directly by a given actor. In the study, the private costs and benefits of layer feedlot owners were analysed (Figure S8).

The formula for calculating the total cost of all variable inputs in laying hens’ production is as follows:

(6) $$\begin{array}{c} LCC_{total}=C_{chick}+C_{feed}+C_{labor}+C_{dp}+C_{we}+C_{contruction}+C_{equipment} \end{array}\;$$

where ${LCC}_{total}$ is the life-cycle cost of layer rearing; $C_{chick}$, $C_{feed}$, $C_{labor}$, $C_{dp}$, $C_{we}$, $C_{contruction}$, $C_{equipment}$ represent, respectively, chick cost, feed cost, labor cost, disease-prevention cost, water and electricity cost, construction cost, and equipment cost.

The gross margin was applied to compare the gross margin from unit cost among three types of farms and is calculated as follows:

(7) $$Gross\;Margin=Gross\;Return-{LCC}_{total}$$

where, Gross Return is the income of the laying hen farm.

## 3. Results

### 3.1. Drivers and Barriers to the Application of LPD Technology

The average size of layer farms in Quzhou county is 11,699 hens, closely resembling the average scale of farms in the North China Plain. The average CP content in the diet for pre-laying, peak-laying, and late-laying hens is 17.3%, 16.8%, and 16.5%, respectively (Figure S9). It is noteworthy that the average CP levels in the early-laying and late-laying stages exceed the national recommended LPD standards by 0.3 and 0.5 percent in absolute value, respectively.

**Figure 2:**
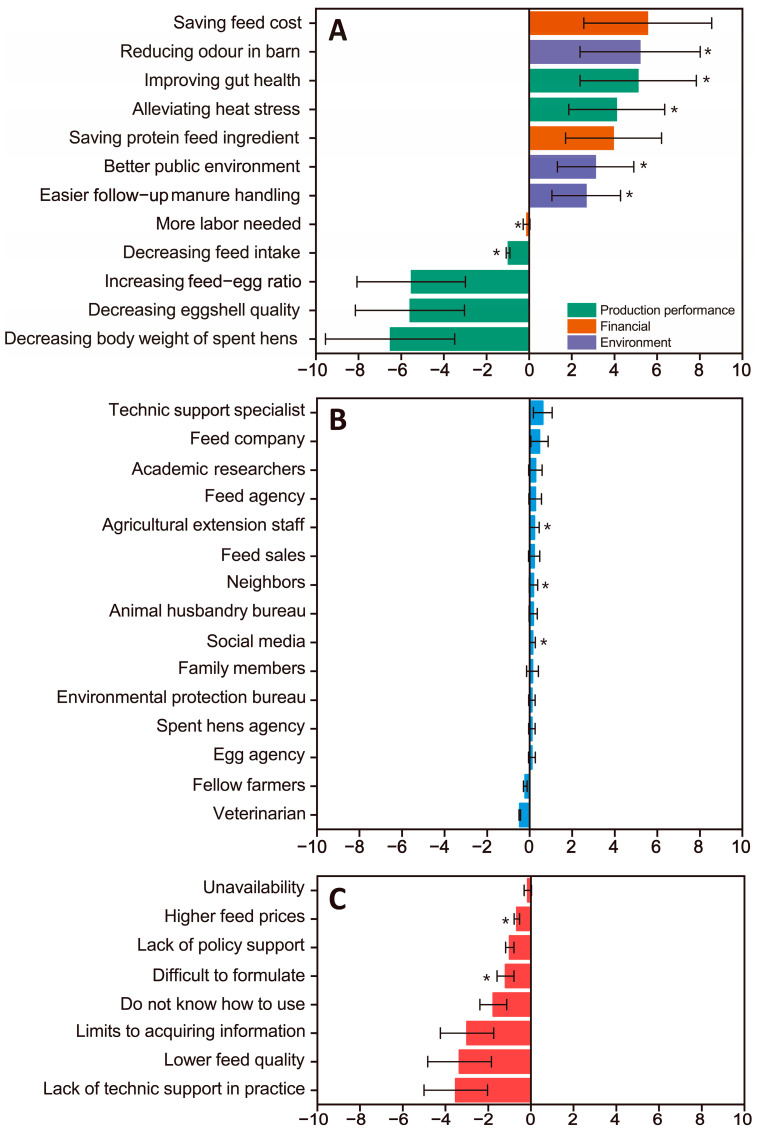
Drivers of and barriers to using low-protein diet, based on results of all farmers surveyed in this study (N = 96; solid bars indicate average scores; the error bars (lines) indicate the standard deviations). Different colors indicate different categories of attitude variables. (**A**): Attitudes; (**B**): Subjective norms; (**C**): Behavioral controls. * Indicates the mean strength score < 3.

Cost-saving and conserving protein feed ingredients are driving factors influencing farmers to adopt LPD technology (Figure 2A). Environmental impacts (e.g., reduced barn odors, improved common areas, and ease of manure disposal) and improved performance (improved gut health and relief of heat stress) are potential drivers (strength of belief < 3). Barriers perceived by layer farmers in adopting LPDs are associated with decreased body weight of spent hens, lower eggshell quality, and increased feed–egg ratio, reflecting a low level of trust in the quality of LPDs (Figure 2A). Despite the significance of feed intake for egg production, especially in the summer, some farmers believe that LPD usage poses a low risk of reducing feed intake (i.e., belief strength < 3; Figure 2A). Labor requirements are also negatively evaluated, but at a relatively low level, and farmers perceive a low risk of causing such a situation (belief strength < 3; Figure 2A).

In the social referents category, all thirteen driving factors and two barrier factors score below 3. Technical service specialists, feed companies, and academic researchers exert a relatively positive influence (Figure 2B), while veterinarians and fellow farmers have a more negative impact. The government sectors such as animal husbandry and environmental protection, agricultural extension staff, social media, and parties along the industry chain including feed suppliers and egg marketers, as well as neighbors and family members, all show limited influence on the adoption of LPDs. Layer farmers perceive three key barriers: (i) lack of practical technical support, (ii) lower feed quality, and (iii) limitations in obtaining information about LPDs. Additionally, layer farmers perceive difficulty in LPD usage in practice, lack of policy support, and unavailability of low-protein feed in the market as barriers. They also perceive the challenges in formulating LPDs and their higher cost relative to normal diets as potential barriers (Figure 2C).

### 3.2. Meta-Analysis of LPD Technology on Egg Productivity, Quality, and Feed Efficiency

**Figure 3:**
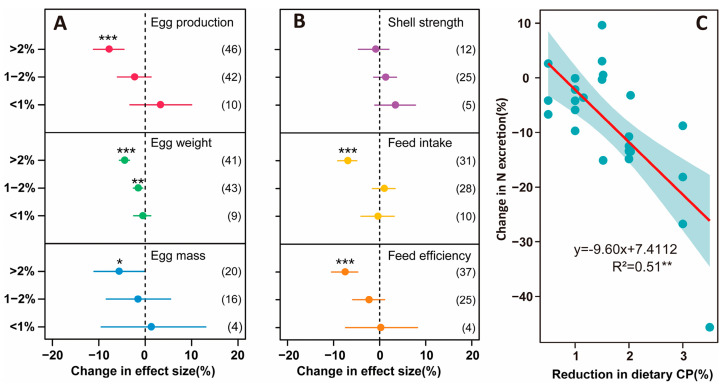
Effects of LPD on (**A**) performance, (**B**) egg quality, and (**C**) environment in laying hens, as function of different dietary CP contents. Points show means of treatments; bars show 95% confidence intervals. Confidence intervals that do not overlap with the zero line indicate statistical significance. The numbers in the parentheses indicate the number of observations on which the statistical analysis was based. The data of Figures (**A**,**B**) are presented in table form in Table S18. Asterisks represent statistically significant differences (* *p* < 0.05; ** *p* < 0.01; *** *p* < 0.001).

The meta-analysis database includes 30 studies, with the average crude protein levels in the control groups reported as 17.33%, 18.06%, and 16.17% for the pre-laying, peak-laying, and late-laying phases, respectively (Table S4). The application of LPD in laying hens can have varying effects on their production performance depending on the extent of protein reduction (Figure 3). A 1% reduction in CP led to a non-significant increase in egg-production rate and eggshell strength, with negligible effects on egg weight, egg production, feed intake, and feed efficiency. When CP reduction reached 1–2%, egg quality decreased significantly by 1.8%, while other indicators remained unaffected. However, reductions exceeding 2% resulted in significant decreases in egg production (8.6%), egg weight (5.0%), feed intake (7.3%), and feed efficiency (7.8%), with minimal impact on eggshell strength. CP in the diet is significantly negatively correlated with N emissions, decreasing as the extent of CP reduction increases (Figure 3C). As CP content decreases, egg- production rate (*p* < 0.01), egg production (*p* < 0.01), feed intake (*p* < 0.01), and feed-conversion rate (*p* < 0.01) all decrease (Figures S4–S6).

### 3.3. N Footprint and Life-Cycle Costs of the Laying Hen Supply Chain with LPD Technology

**Figure 4:**
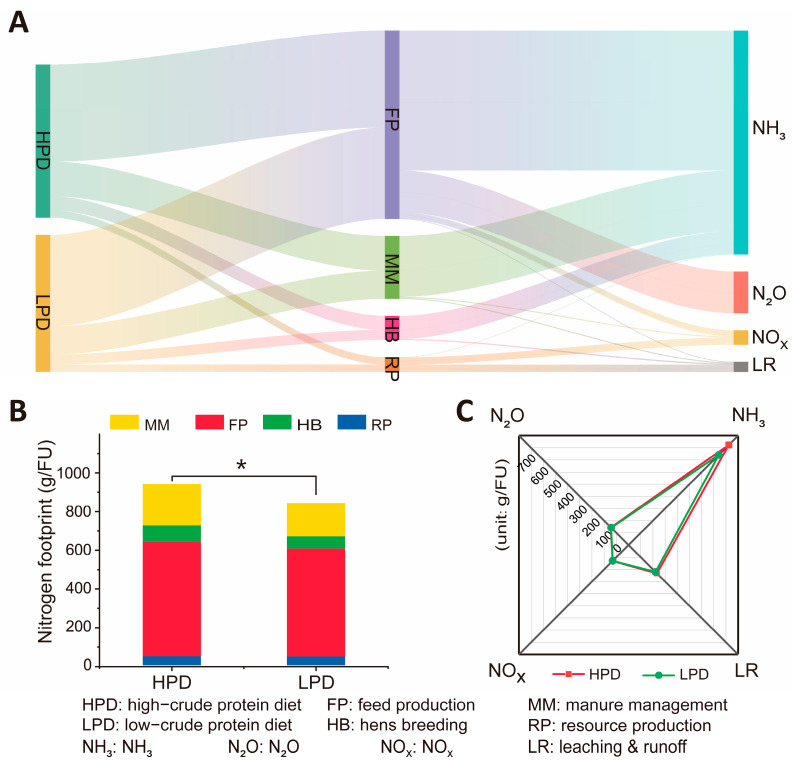
Nitrogen footprint under different CP contents in the daily feed. (**A**): Varied gas emissions at different production stages. (**B**): Nr loss at different stages. (**C**): Various forms of Nr loss. * Indicates significant differences between the two (*p* < 0.05). Functional unit (FU) is a carton of eggs (18 kg).

The N footprint of egg production chain primarily occurred in feed production and manure management, with NH_3_ and N_2_O being the main forms of loss (Figure 4A). Compared to farmers who used high-crude protein diets (HPD), the use of LPD technology significantly reduced N footprint (*p* < 0.05, Figure 4B). In the entire system, the total Nr loss for farmers using HPD was 932.89 g/FU. The distribution ratio of Nr losses at each stage were as follows: resource production, 4.90%; feed production, 63.31%; manure management, 22.65%; and hens breeding, 9.13%. For LPD farmers, the total Nr loss was 835.40 g/FU, and the contribution of N footprint at each stage were as follows: resource production, 5.28%; feed production, 66.80%; hens breeding, 7.43%; and manure management, 20.48%.

Farmers who use the LPD technology not only reduced N footprint but also increased profit (Table 1). Since only the CP content of feed is reduced, costs for chick rearing, water and electricity consumption, disease prevention and control, and housing remain largely unchanged. However, adopting LPD technology by farmers can increase profits by 1.15 times when compared with high-protein diets (HPDs). Farmers that used LPDs instead of HPDs raised their egg sales income by 8.78 CNY bird^−1^ cycle^−1^ due to lower feed costs. In addition, farmers using LPD technology had a 116% higher cost-effectiveness ratio than those using HPDs.

## 4. Discussion

### 4.1. Why Farmers Do Not Use LPDs and How to Motivate Them to Use LPDs

LPD technology is acknowledged for its substantial environmental and economic benefits [36,37]. Moreover, under the condition of reasonable amino-acid supplementation, the production performance of laying hens is not adversely affected [38]. Despite these advantages, farmers often perceive weight loss in chickens, a decrease in eggshell quality, and an increase in the feed–to–egg ratio as negative outcomes of and obstacles to LPD technology implementation (Figure 2). These perceptions stem from the belief that protein is essential for growth, and reducing protein content will harm production performance. Additionally, the inconsistent quality and higher price of low-protein feed in the market further contribute to these perceptions. Research indicates that anticipated economic returns are the primary motivation for investing in certified livestock facilities [39]. There is also the possible factor that farmers do not have the conditions or basis for supplementing layers’ diets with amino acids, which can lead to nutritional imbalances, quality loss, and other problems. Perceived risks, particularly financial ones, pose significant obstacles to adopting new technologies [40]. It can be observed that the primary demands of farmers for technological application are to increase yield, ensure production performance, and enhance profitability.

In practical application, laying hen farmers face significant obstacles, including lack of technical support, restricted access to LPD information, and uncertainty about effective LPD usage (Figure 2). The complexity of adopting agricultural technology is influenced by risk preferences, human capital, and cooperative membership [41,42,43]. In this study, the majority of egg farmers lacked practical experience with LPD technology (Table S2). This is due to their lack of knowledge or relevant information about LPD technology, and an inability to seek guidance from third parties such as government agencies, academic institutions, and feed companies. Additionally, there is a significant positive correlation between the willingness to use LPD technology and the age of egg farmers (Figure S10), with older farmers being less inclined to adopt the technology. The study finds that aging significantly affects the sustainable development of agriculture in China [44], as older farmers generally have lower levels of education and a reduced willingness to embrace new technologies. This finding underscores the importance of designing customized training and support programs for farmers of different age levels when promoting new technologies. Through increased education and technical support, the rate of adoption of new technologies can be significantly increased, thereby contributing to sustainable agricultural development. Zhao et al. [45] noted that small-scale farmers who underwent training twice a year from 2009 to 2013 experienced improved maize yields and N-use efficiency. Therefore, enhancing awareness and willingness among farmers to use LPDs can be achieved through strengthened promotion and training. Furthermore, the study identifies a lack of policy support as another obstacle to the adoption of LPD technology. Issues experienced in technological transformation are linked to the absence of government and institutional support [46]. In this research, farmers were unable to access financial aid or other incentive measures, resulting in limited motivation for adopting this technology (Figure 2). Thus far, government policies have primarily focused on the implementation of LPDs by large corporations in a ‘top-down’ system. Its advantages are: (1) professional nutritionists to support the technical realization; (2) quality control of raw materials and low-protein feeds; and (3) covering a wide range of customer groups, especially for large-scale farmers. Governments can support the diffusion of LPD technology by providing financial incentives, technology training programs, and promoting cooperation between governments and enterprises. For example, governments can subsidize farmers who adopt LPD technology or fund technical training programs to enable farmers to better understand and apply the technology. In addition, encouraging cooperation between feed companies and research institutions to develop high-quality, low-cost low-protein feed products can also promote the spread of this technology.

Social reference has limited influence on the intention of small-scale farmers to adopt LPDs, whether positively or negatively (Figure 2B). Most farmers perceive these social references as ‘indifferent’ toward their use of LPD, and when asked if they would follow the recommendations, the response is generally ‘depending on the situation’ (Figure S11). The majority of egg chicken farmers are unaware of LPD technology. Additionally, they lack an understanding of the importance of social agents in the dissemination of new technologies. This reflects that the advanced technology has not been widely recognized and accepted by stakeholders in the egg industry or government departments. The ‘top-down’ dissemination system for LPD technology has not been fully established. Farmers’ decisions are influenced by social norms in various ways [47]. Research scholars formulate and plan new technologies based on theoretical foundations, while agricultural extension advisors from government departments (such as the Livestock Bureau and Environmental Protection Bureau) facilitate the transfer of these technologies to farmers [48]. However, most surveyed farmers show higher levels of trust in social references with whom they spend the most time (such as technical service experts and veterinarians) (Figure 2). Interestingly, the influence of these two stakeholders on farmers’ use of LPDs is different. In rural areas, providers of feed and disease services play a crucial role in reshaping farmers’ decisions about LPD technology. Therefore, establishing trust among these intimate stakeholders is essential in rural areas. In addition, government subsidies and farmers’ awareness of cost risks will affect the willingness to use the technology [49].

Apart from businesses and technical service experts, other social factors such as research experts, agricultural extension personnel, and local community factors show no significant impact on the promotion of LPD technology (Figure 2). This partly reflects the disconnect between research and production and the lack of societal factors driving a reasonable understanding of LPD technology. There is a need to strengthen the popularization and promotion of the technology. A survey was conducted with 96 farmers, including 20 who had received training in LPD technology (Section 2.1.2). The results show that training is essential for farmers to understand and adopt the new technology, as it increases their awareness of LPDs and their willingness to use them. Therefore, efforts should be made to enhance the proactiveness of research institutions and universities in transforming achievements, adopt a market-oriented approach, strengthen collaboration between businesses and research institutions, promote technology, and create a new situation where enterprises, research institutions, and universities are organically coordinated.

### 4.2. Opportunities and Trade-Offs Among Environmental and Economic Performances in Applying LPDs

China is facing a shortage of feed protein and is heavily relying on imports, making the adoption of LPD technology particularly urgent [50,51]. This technology can significantly reduce N footprint, especially NH_3_ emissions (Figure 3C), playing a positive role in mitigating environmental pollution and maintaining the ecological environment around farms. The mechanism of LPDs primarily involves regulating protein levels, reducing N intake in laying hens, and increasing N-use efficiency, effectively reducing NH_3_ emissions and mitigating adverse impacts on the environment [36]. However, protein plays a crucial role in feed. Improper control may negatively affect the growth, development, and production performance of laying hens (Figures S4 and S5). Research results indicate that a 1% reduction in low-protein feed has no significant impact on production performance (Figure 3A,B), consistent with findings from other studies [52]. However, exceeding this proportion may have certain effects on the production performance of laying hens, such as egg production rate and hatchability [53]. Therefore, a careful balance between reducing protein intake and maintaining production performance is necessary to ensure the overall stability of the flock [54]. However, this is relatively challenging for farmers. It is essential to ensure the provision of sufficient amino acids and trace elements to compensate for the deficiencies in low-protein feed [53]. In feed formulations, the types and amounts of amino acids should be fully considered to ensure production performance [38]. Moreover, these formulations should be prepared in advance to reduce the difficulty of technology use. Relatively simple technologies increase farmers’ willingness to use them [55]. Through precise formulation and optimization of protein supplementation, more economical feed utilization can be achieved [54]. In the total cost of animal production, feed costs account for over 70%, exceeding the total output [56]. Any factors that increase feed costs may lead to reduced farmer income, forcing them to optimize feed use to maintain the profitability of the production system [57]. LPD technology happens to increase farmers’ incomes without increasing feed costs (Table 1), providing an economically viable option for farmers.

**Figure 5:**
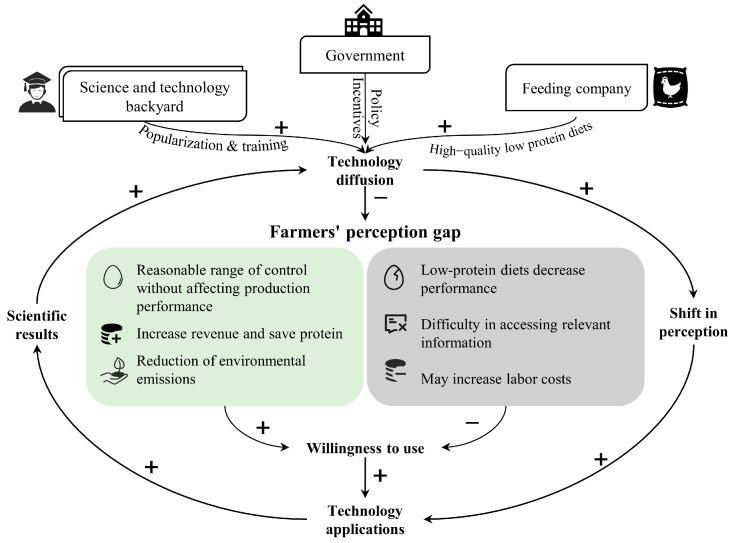
Framework for the application and diffusion of LPD technology. An illustration of the framework to dispel farmers’ perception gaps through the promotion of low-protein diet technologies. The arrows represent a causal relation between two factors, and the polarity of an arrow indicates whether the relation is positive or negative.

In summary, to promote the widespread application of LPD technology, it is essential to strengthen technical training and support for farmers, enhancing their understanding and confidence in this technology (Figure 5). Strengthening these collaborations and increasing the frequency and quality of training sessions can significantly enhance the adoption of technology [58]. In China, graduate students in Science and Technology Backyard (STB) who live in villages or near farmers are well positioned to spread these advanced technologies [59]. When formulating policies, the government should pay more attention to the needs of grassroots farmers, providing more incentives and support to reduce the financial risks of adopting new technologies. Additionally, communication and trust-building with farmers should be strengthened to ensure smooth technology dissemination. In the process of technology development, a comprehensive consideration of the production performance of laying hens and environmental protection is necessary. Through precise formulation and nutritional supplementation, the practical application of LPD technology can yield more significant results.

## 5. Conclusions

This study elucidated the insufficient understanding of LPD technology among small-scale farmers and the obstacles to its adoption, and also developed a promotion route for LPD technology in small- and medium-sized egg chicken farms. Through a farmer perception survey, it was found that 84% of the farmers lacked understanding of LPD technology and its nutritional value, and there were cognitive misunderstandings. However, a meta-analysis revealed that under reasonable control conditions, LPDs had no significant negative effects on laying hen performance and significantly reduced N excretion (each 1% reduction in CP reduces N excretion by 9.6%). LCA and LCC analyses also showed that LPDs provide environmental benefits as well as increased economic benefits. The reason for the discrepancy between farmers’ perceptions of LPDs and their actual effects is partially related to the lack of effective communication between governmental departments, research institutes, feed companies, and farmers. Therefore, the study suggests that the government should formulate science-oriented policies to promote cooperation among stakeholders. At the same time, technical training and publicity for farmers should be strengthened to promote the wide application of LPD technology in livestock production. The technology promotion framework of this study is not only of great significance to China, but also has important reference value globally, especially for developing countries facing challenges such as protein feed shortages and environmental pollution.

## Figures and Tables

**Table 1:** Inventory for LCC, revenue, and profitability analysis of HPD and LPD farmers (CNY bird^−1^ cycle^−1^).

Items		Farmers Using HPD (n = 91)		Farmers Using LPD (n = 5)	
		Total Price	Percentage	Total Price	Percentage
Life- Cycle Cost	60-day-old chicken cost	15.00	8.51	15.00	8.53
	Water and electricity cost	2.25	1.28	2.25	1.28
	Disease prevention cost	3.00	1.70	3.00	1.71
	Construction cost	6.00	3.40	6.00	3.41
	Feed cost	150.10	85.11	149.66	85.08
	Total cost	176.35	100	175.91	100
Revenue	Eggs	160.34		169.12	
	Spent hens	24.00		24.00	
Profitability	Profit	8.00		17.21	
	Cost–benefit ratio	4.53		9.79	

Note: HPD indicates a higher dietary crude protein content, which is the traditional practice by local farmers. LPD indicates a lower crude protein content. Reduction in crude protein content ranged from 0.36% to 0.79%.

## Data Availability

The data presented in this study are available on request from the corresponding author.

## Supplementary Materials

The following supporting information can be downloaded at: https://www.mdpi.com/article/10.3390/ani15020146/s1, Figure S1: Illustration of the framework of this study, following the Theory of planned behavior approach; Figure S2: Distribution of investigated layer farms in Quzhou County; Figure S3: Study selection process. A total of 807 studies were retrieved from the electronic databases (Web of science and China National Knowledge Infrastructure). Finally, a total of 30 studies were included in the study; Figure S4: Effects of low-protein diet on egg production (a), egg weight (b) and egg mass (c) in laying hens; Figure S5: Effects of low-protein diet on feed intake (a) and feed efficiency (b) in laying hens, as function of different laying periods (pre-laying, laying peaking, late laying and overall periods: see definitions in the main text); Figure S6: Relation between reduction in dietary CP content and change in egg production (a), egg weight (b), feed intake (c) and feed efficiency (d), expressed as a percentage of the reference treatment; Figure S7: LCA System boundary of layer production; Figure S8: LCC System boundary of layer production; Figure S9: Dietary CP content of laying hens for farms surveyed (N = 94). Orange point and light green point are limit superior and inferior of national standard for each phase of laying hens, respectively; Figure S10: Intention scores of farmers for using low-protein diet during the next three years (N = 96); Figure S11: Spearman’s rank correlations between the intention of layer farmers to use LPD and the farm and farmers’ characteristics. Table S1: Layer farm scale distribution in this study; Table S2: Characteristics of surveyed layer farms in Quzhou county; Table S3: Average feed formula of laying hens in this study; Table S4: Mean values of CP content and its range of reduction in the control group.
