# Supplementary material for: Integrated Assessment of Productive, Environmental, and Social Performances of Adopting Low-Protein Diets Technology for Laying Hens
Source: Animals (Basel). 2025 Jan 9;15(2):146. doi: 10.3390/ani15020146 (PMC11758341; doi:10.3390/ani15020146)
Supplement: Supplementary file 1 [file animals-15-00146-s001.zip › animals-3380534-supplementary.pdf]

Integrated Assessment of Productive, Environmental, and Social Performances of Adopting Low-Protein Diets Technology for Laying Hens

Dongsheng Li [a], Xiaoying Zhang[a], Siqi Wang[a], Zhiyang Zhao[a], Jing Wang[b], Hongliang Wang[a,*]

[a] *State Key Laboratory of Nutrient Use and Management, College of Resources and Environmental Sciences, China Agricultural University, Beijing 100193, China*

[b] *Institute of Feed Research, Chinese Academy of Agricultural Sciences, Beijing 100081, China*

*Correspondence: Hongliang Wang, China Agricultural University, Beijing 100193, China. (E-mail: wang_hl@cau.edu.cn).

This supplemental information includes 18 tables, 11 figures, and detailed methods for calculating nitrogen losses.

A total of 11 supplementary figures, as follows:

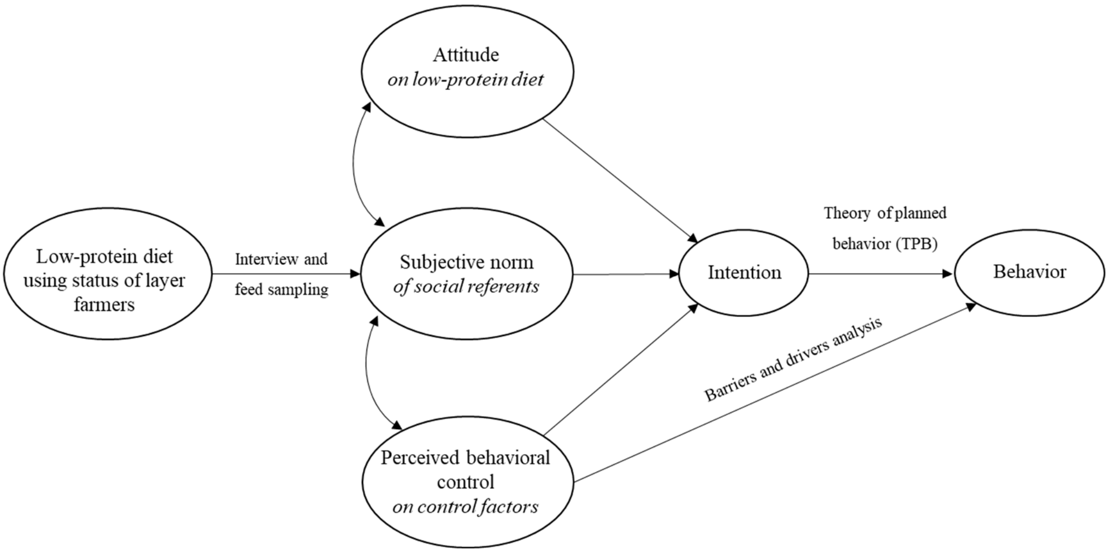

Figure S1. Illustration of the framework of this study, following the Theory of planned behavior approach (Ajzen,2011).

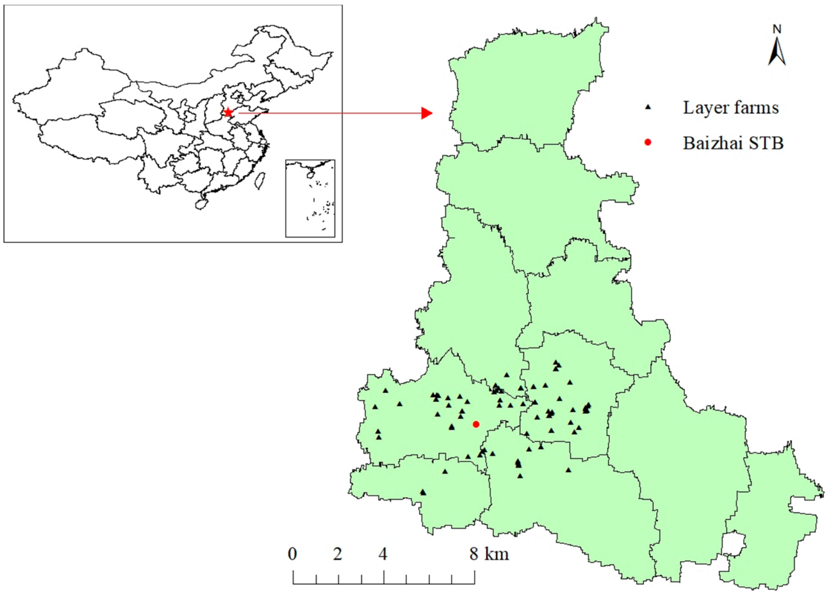

Figure S2. Distribution of investigated layer farms in Quzhou County.

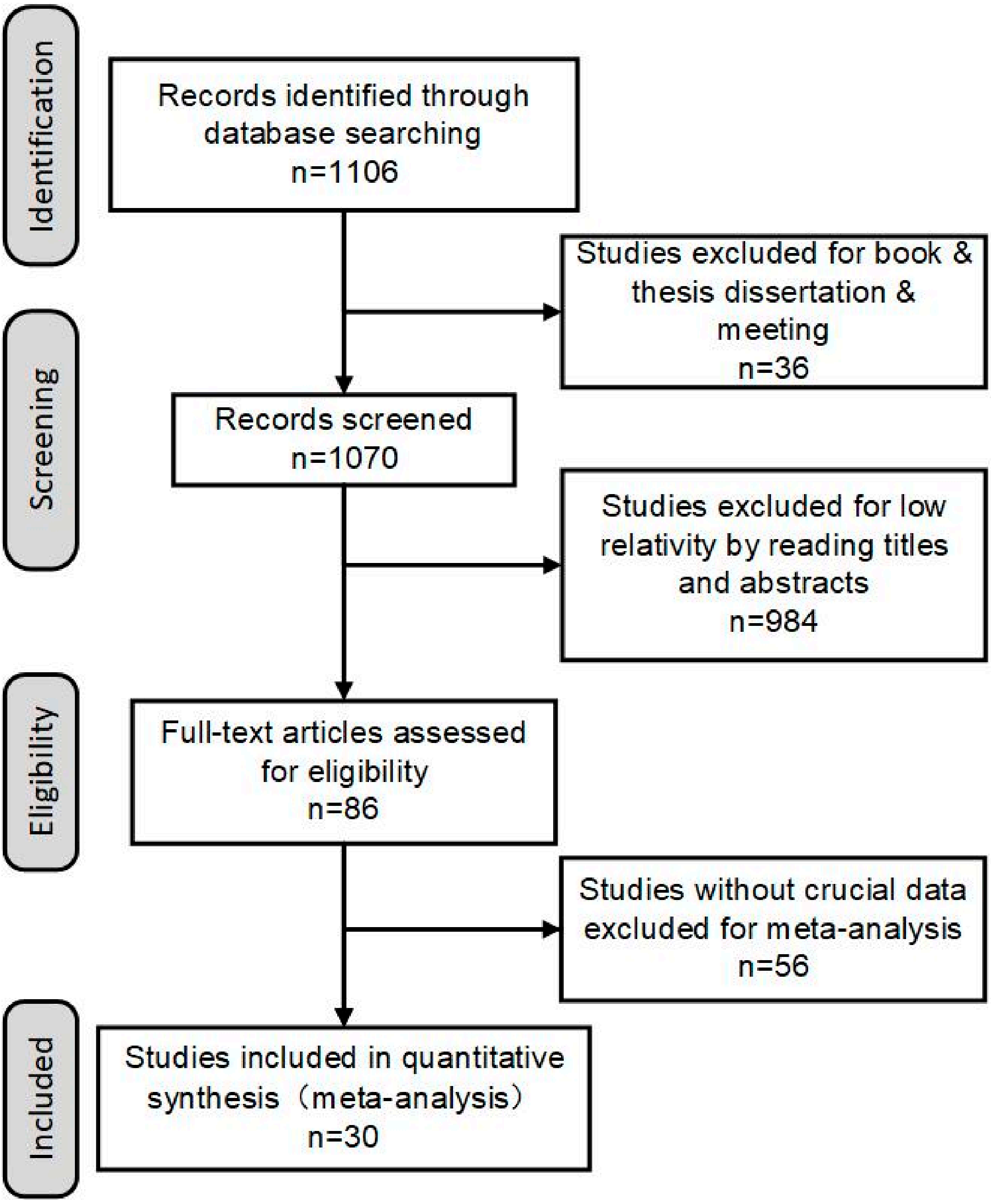

Figure S3. Study selection process. A total of 807 studies were retrieved from the electronic databases (Web of science and China National Knowledge Infrastructure). Finally, a total of 30 studies were included in the study.

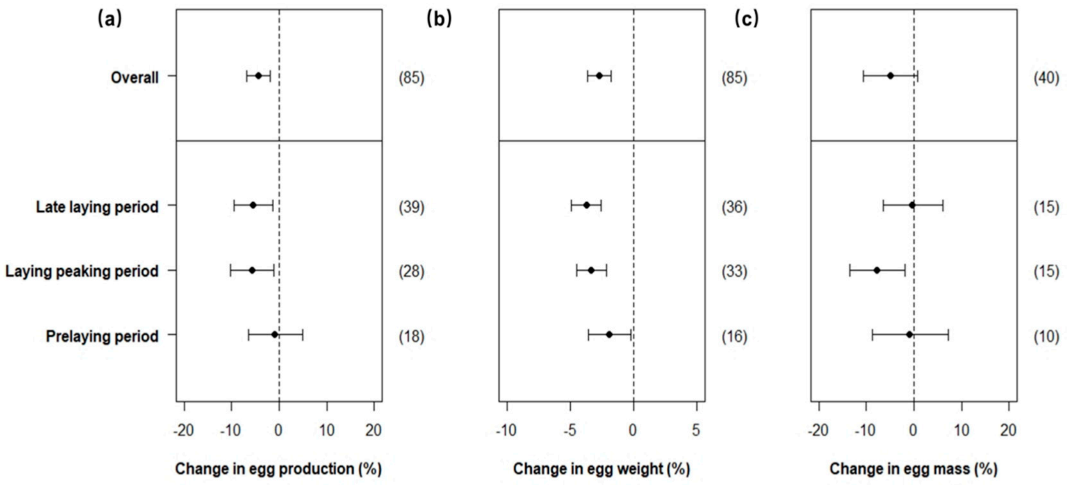

Figure S4. Effects of low-protein diet on egg production (a), egg weight (b) and egg mass (c) in laying hens, as function of different laying periods (pre-laying, laying peaking, late laying and overall periods: see definitions in the main text). Points show means of treatments, bars show 95% confidence intervals. Numbers in the parentheses indicate the number of observations on which the statistical analysis was based.

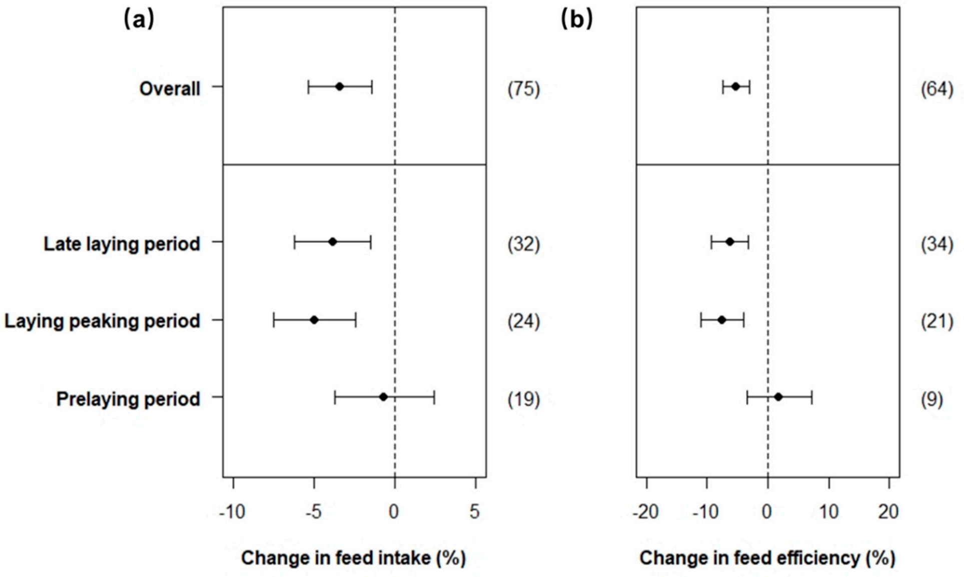

Figure S5. Effects of low-protein diet on feed intake (a) and feed efficiency (b) in laying hens, as function of different laying periods (pre-laying, laying peaking, late laying and overall periods: see definitions in the main text). Points show means of treatments; bars show 95% confidence intervals. Numbers in the parentheses indicate the number of observations on which the statistical analysis was based.

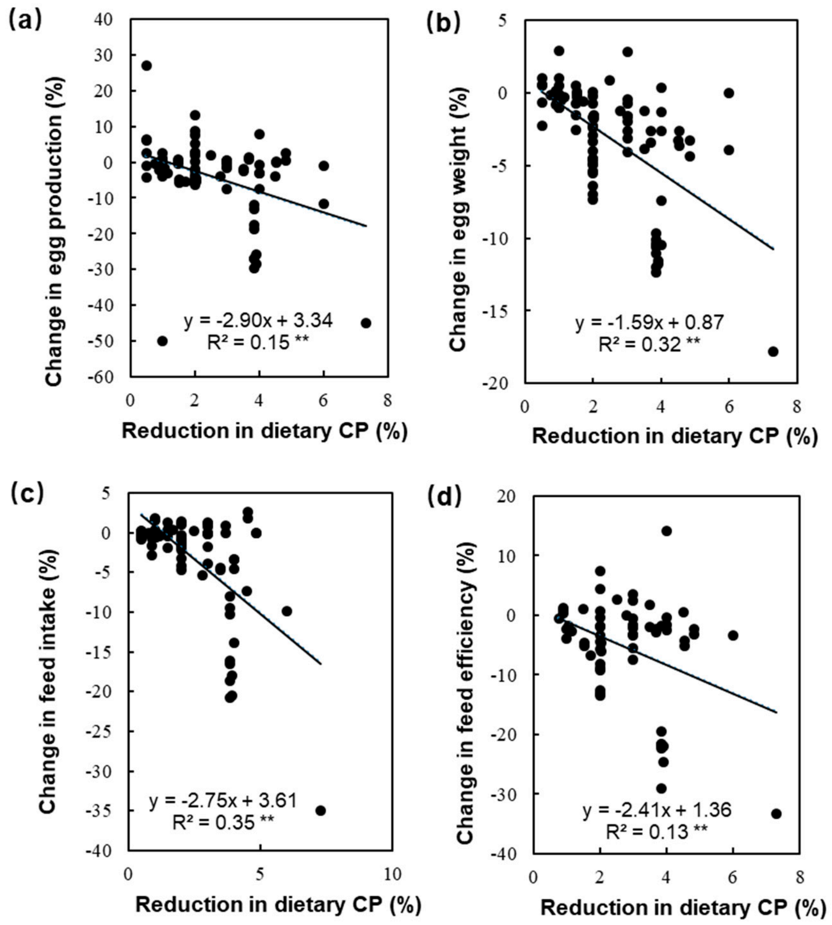

Figure S6. Relation between reduction in dietary CP content and change in egg production (a), egg weight (b), feed intake (c) and feed efficiency (d), expressed as a percentage of the reference treatment. Significance asterisks ** = $P < 0.01$.

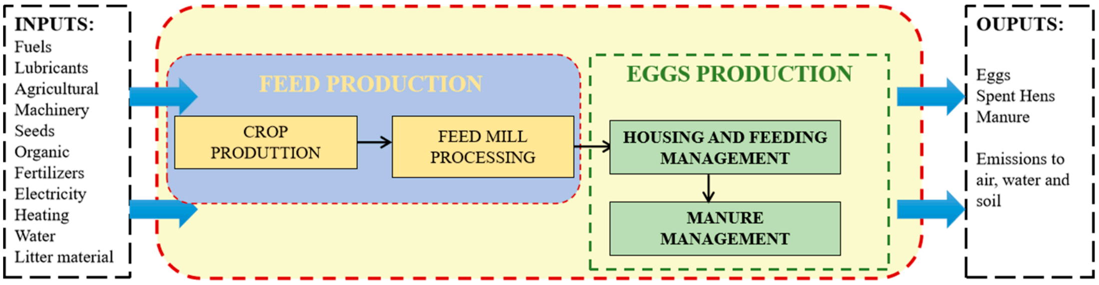

Figure S7. LCA System boundary of layer production.

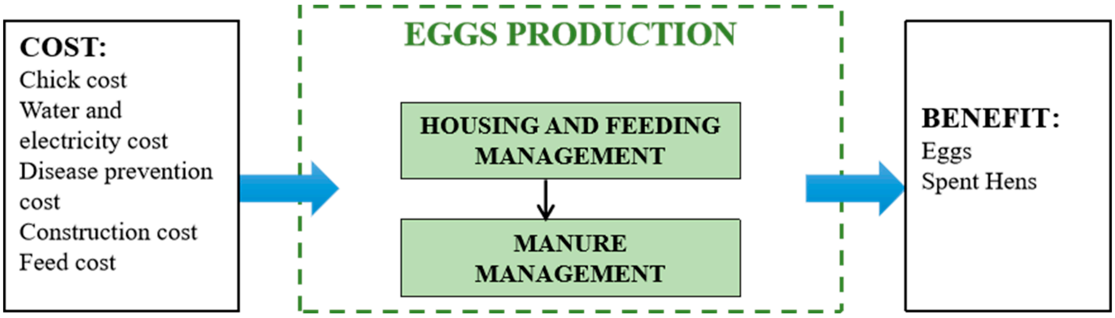

Figure S8. LCC System boundary of layer production.

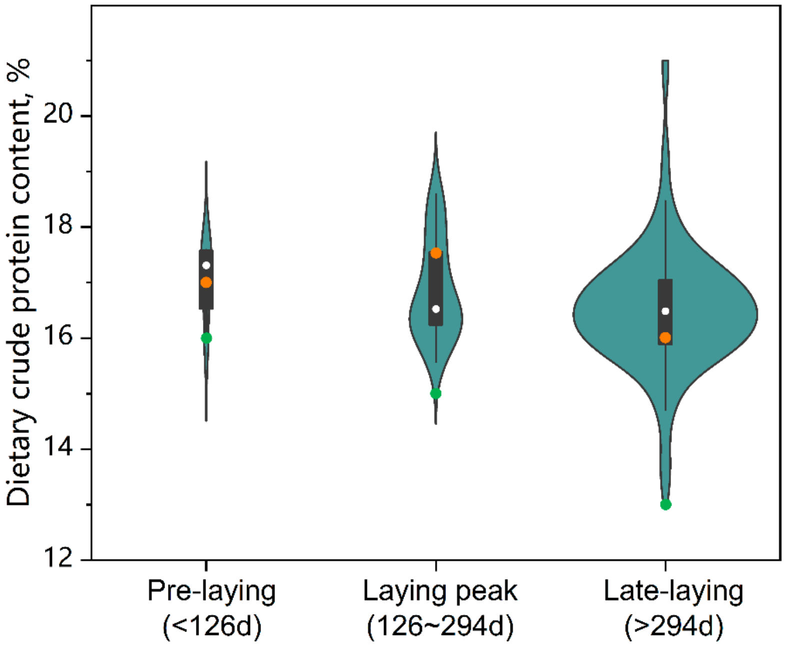

Figure S9. Dietary CP content of laying hens for farms surveyed (N=94). Orange point and light green point are limit superior and inferior of national standard for each phase of laying hens, respectively.

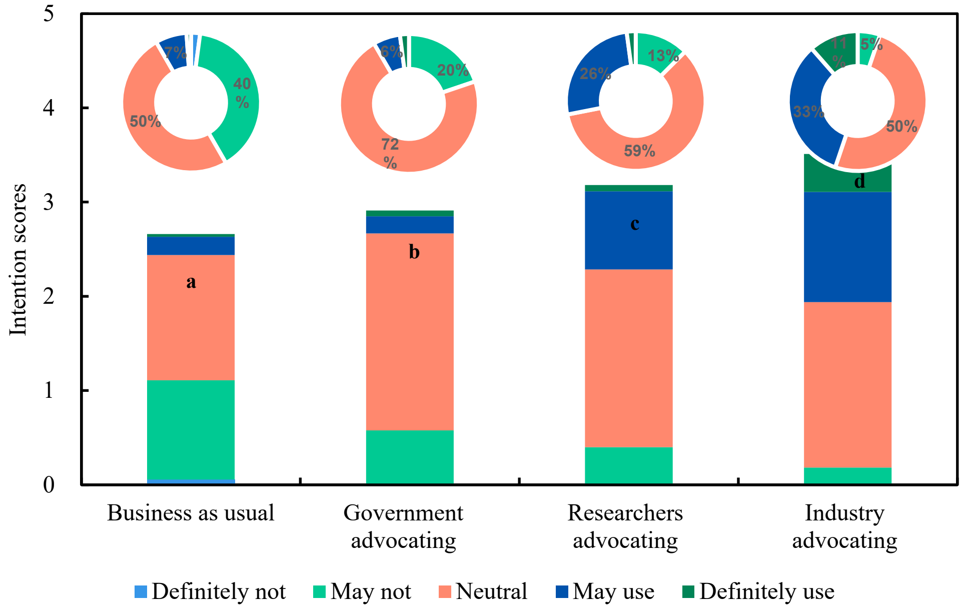

Figure S10. Intention scores of farmers for using low-protein diet during the next three years (N = 96). Results for using low-protein diet by layer farmers expressed on a Likert scale of 1 to 5. Pie charts indicate relative proportion of responses. Different letters above the bars indicate significant differences between scenarios.

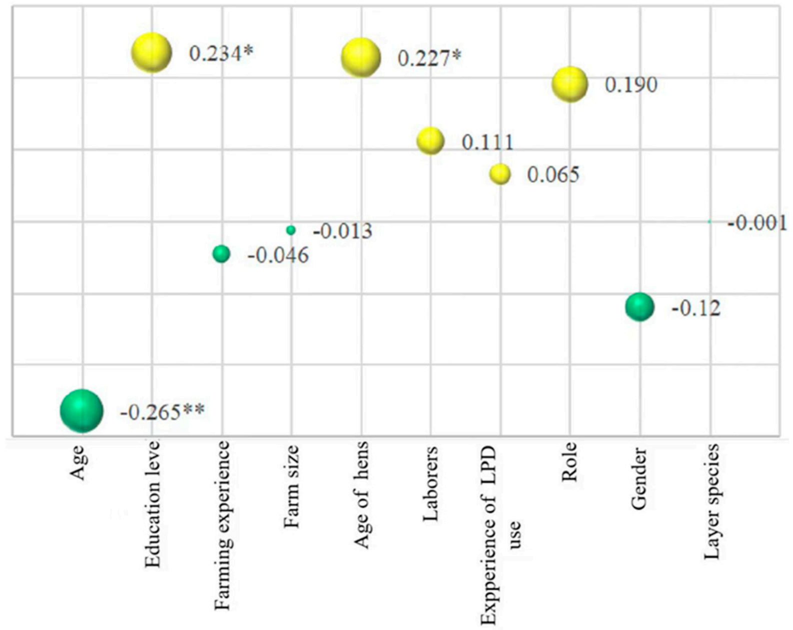

Figure S11. Spearman's rank correlations between the intention of layer farmers to use LPD and the farm and farmers' characteristics. Numbers indicate Spearman's rho. *Indicates the significance of the coefficients at P <0.05, ** indicates significance at P <0.01. Larger sphere sizes indicate a higher Spearman's rho. Colors indicate correlation direction (the light yellow indicates positive; the light green indicates negative).

A total of 18 supplementary tables, as follows:

Table S1. Layer farm scale distribution in this study.

Scale (hens per farm)	Farms		Hens housed		Cumulative (%)
	Number	%	Number	%	
<5,000	6	6.3	24700	2.5	2.5
5000-9999	40	41.7	285100	28.3	30.7
10,000-2,9999	43	44.8	558300	55.4	86.1
≥30,000	7	7.3	140000	13.9	100

Data source: survey data.

Table S2. Characteristics of surveyed layer farms in Quzhou county.

Items	Characteristics
Number of farms surveyed	96
Stock number of laying hens	11,699 (4,000-45,000)
Age of laying hens	336 (70-700)
Layer species	
Roman series, %	6%
Hy-Line series, %	25%
Jing Tint series, %	48%
Dawu Jinfeng, %	18%
Nongda series, %	3%
Age of the interviewees (years old)	46
Role of the interviewees	
Farmer, %	89%
Cottar, %	11%
Gender of the interviewees	
Male, %	73%
Female. %	27%
Education of the interviewees (level)	2.6
Experience in egg production of the interviewees (years)	10.8
Number of workers on the farm (persons)	1.8
Experience on low-protein diet use of the interviewees	
No experience, %	84%
Minor experience, %	16%
Experienced, %	0%

Data source: survey data.

Table S3. Average feed formula of laying hens in this study.

Feed components, %	Physiological Stage		
	Pre-laying period	Laying peaking period	Late laying period
Maize	62~65	62~68	60~70
Soybean meal	21~25	20~25	20~30
Wheat bran	2.5	0.3~4	0~2
Soybean oil	0.25~0.5	0.3~0.5	0.25~1
Stone powder	4~8	2~9	2~8
Premix	5	2~5	2~5

Data source: survey data.

Table S6. Characterization of the manure management systems (%).

Characteristics	Farmers using HPD (N=91)	Farmers using LPD (N=5)
Type of floor		
Conveyer	40	0
Slatted floor	60	100
Cleaning frequency		
Once in 2 or more days	88	100
Once a day	12	0
Manure storage		
Covered and underground	11	0
Covered and aboveground	0	0
Uncovered and underground	80	0
Uncovered and aboveground	9	100
Manure treatment		
Anaerobic fermentation	0	0
Household biogas	0	0
Industrial biogas	0	0
Composting	30	33.3

Note: HPD indicates a higher dietary crude protein content, which is the traditional practice by local farmers. LPD indicates a lower crude protein content. Reduction in crude protein content ranged from 0.36% to 0.79%. Data source: survey data.

Table S5. Mass ratio and economic allocation of co-products from different feed crops (%)[a].

Feed crop	Co-products	Mass ratio	Economic allocation
Wheat	Wheat grain	39	77
	Wheat straw	61	23
Wheat grain	Wheat bran	12	6
	Wheat flour	74	84
	Wheat germ	2	3
	Wheat middling	12	7
Soybean	Soybean meal	71	56
	Soybean oil	22	41
	Soybean hull	7	3

[a]:(Nemecek et al., 2007), (Long et al., 2021).

Table S4. Mean values of CP content and its range of reduction in the control group.

	All trials (%)		Trials with ≤1% reduction in CP			Trials with >1% reduction in CP		
Stage	Control	*N*	Control	*N*	Reduction in treatments	Control	*N*	Reduction in treatments
Pre-laying	18.78 (15.37~21.62)	12	17.33 (16.00~18.00)	6	0.75 (0.5~1.0)	18.43 (15.37~21.62)	20	2.45 (1.5~4.4)
Laying peak	17.72 (15.37~20.10)	21	18.06 (16.50~20.10)	10	0.77 (0.5~1.0)	17.60 (15.37~19.44)	28	2.75 (1.5~7.3)
Late-laying	16.86 (15.00~21.88)	29	16.17 (16.00~16.50)	7	0.83 (0.5~1.0)	17.00 (15.00~21.88)	36	2.86 (1.1~6.0)

Note: the numbers in parentheses indicate the maximum and minimum values of dietary protein content (or reduced level of crude protein compared to the control group) under that category. The letter N represents the number of controls in the original study database.

Table S7. Emission factors of Nr from the production of various agricultural inputs.

Agricultural inputs	Unit	N_2O emission ($g\ N\text{-}N_2O\ unit^{-1}$)	NO_x emission[a] ($g\ N\text{-}NO_x\ unit^{-1}$)	Reference
Nitrogen fertilizer	kg N	0.09	13.47	(Liang., 2009), (Zhang et al., 2013)
Phosphorus fertilizer	kg P_2O_5	0.013	2.16	(Chen et al.,2015), (Xia et al., 2016)
Potash fertilizer	kg K_2O	0.017	2.9	(Chen et al.,2015), (Xia et al., 2016)
Compound fertilizer	kg	0.03	4.49	(Long et al., 2021)
Agricultural film	kg	0.19	15.1	(Wang et al.,2015), (Xia et al., 2016)
Pesticide	kg	0.18	14.3	(Yang et al., 2014), (Xia et al., 2016)
Maize seed	kg	N/A	N/A	(Thomas et al., 2015)
Wheat seed	kg	N/A	N/A	
Soybean seed	kg	N/A	N/A	
Coal	kg	N/A	2.28	(China energy statistical yearbook, 2016), (Jiang., 2016)., (Li et al., 2016), (Yang et al., 2018)
Diesel	kg	N/A	9.43	(Jiang., 2016)., (Li et al., 2016), (Yang et al., 2018), (Ma.,2002)
Electricity	kWh	N/A	1.01	(Zhang et al., 2013), (Jiang., 2016)., (Li et al., 2016), (Yang et al., 2018)
Steam	MJ	N/A	0.28	(Thomas., 2015), (Jiang., 2016)., (Li et al., 2016), (Yang et al., 2018)

[a] It is assumed that the NOx released by the combustion of fossil fuels from energy consumption is released in the form of NO_2 (Long et al., 2021). N/A means no data.

Table S8. Emission factors of transport (g/km/t).

Transport type	NH_3	Reference
Truck transport	0.026	(Huang et al., 2012), (Zhou, 2018), (Du et al., 2018),
Train transport	0.026	(Nemecek et al.,2007), (Zhang et al., 2013)

Table S9. Straw to grain ratio of feed crop cultivation[a].

Item	Straw to grain ratio
maize straw: maize	1.2:1
wheat straw: wheat	1.1:1
Soybean straw: soybean	1.6:1

[a]:(Ma et al., 2022)

Table S10. Straw recycled back to the field ratio of feed crop production (%)[a].

Item	Straw recycled back to the field ratio
Maize	32.9
Wheat	40.1
Soybean	16.5

[a]:(Ma et al., 2010), (Ma et al., 2012)

Table S11. Nitrogen deposition, biological nitrogen fixation (BNF) of feed crop production[a].

Feed cop	Nitrogen deposition (kg N/ha)	BNF (kg N/ha)	Phosphorus deposition (kg P/ha)
Maize	22.0	18.8	2.75
Wheat	22.0	18.8	2.75
Soybean	22.0	75.0	2.75

[a]:(Ma et al., 2010), (Ma et al., 2012), (Ma et al., 2022).

Table S12. Irrigation nitrogen, Seed nitrogen of feed crop production[a].

Feed cop	Irrigation nitrogen (kg N/ha)	Seed nitrogen (kg N/ha)
Maize	5.0	0.5
Wheat bran	5.0	0.5
Soybean meal	5.0	0.5

[a]:(Ma et al., 2010), (Ma et al., 2012), (Long et al., 2021).

Table S13. Resource use of market feed production [a].

Feed type	N fertilizer (kg N ha^{-1})	Yield (kg ha^{-1})	Plastic film (kg ha^{-1})	Pesticides (kg ha^{-1})	Diesel (kg ha^{-1})[b]	Electricity (kwh ha^{-1})[c]
maize[a]	229.8	7523	5.25	3.79	81.7	350.5
Wheat	237.4	6353.1	0	5.07	95.03	663.2
Soybean	58.1	2100	0	281.3	57.5	57.7

[a]:(NDRC, 2018).

Table S14. Nutrient contents, crop growth period, and root-shoot ratio of main feed crop cultivation [a].

Feed crop	DM (%)	N (% DM)	Ash (% DM)	P (% DM)	C (% DM)	Root-shoot ratio (%)
Maize	87	1.44	2	0.28	57	0.18
Maize straw	80	0.8	7	0.19	54	N/A
Wheat bran	90	2.48	4.8	0.92	55	0.23
Wheat straw	91	0.93	8	0.08	53	N/A
Soybean meal	89	8.24	6.5	0.75	54	0.24

[a]:(China Feed Database, 2020), (Ma et al., 2010), (Wang, 2019), (Liu et al., 2013a); (Li, 2019), (Ma et al., 2022). N/A means no data.

Table S15. The relative partitioning of the layer body and its mean N content (%)[a].

Item	Meat	Bone	Other parts	Egg
Partitioning of layer body	65	20	15	
N content	2.7	2.6	1.5	2.0

[a]:(Ma et al., 2010), (Ma et al., 2012).

Table S16 Emission factors of Nr from fertilizer application and other farmland nitrogen[a].

Emission Factors	Value	Unit
NH_3-N losses from synthetic fertilizer	25	% NH_3-N kg^{-1} applied N
N_2O-N losses from synthetic fertilizer	0.86	% N_2O-N kg^{-1} applied N
NO_x-N losses from synthetic fertilizer	10	% NO_x-N kg^{-1} released N_2O-N
N lost through runoff	9.6	% NO_3-N kg^{-1} applied N
N lost through erosion	0.3	% NO_3-N kg^{-1} applied N
N lost through leaching	18.8	% NO_3-N kg^{-1} applied N

[a]:(IPCC, 2006), (Ma et al., 2010), (Wei et al., 2018).

Table S17. Nitrogen emission factors of manure management and manure application (%)

Item	NH_3-N	N_2O-N	N_2-N	NO_x	Runoff	Leaching	Erosion	Reference
Indoor storage								
Conveyer	7.5	0	0	0	0	0	0	(Wang et al.,2017), In this study
Slatted floor	9	0	0	0	0	0	0	
Outdoor storage								
Permeable without coverage	25	0.5	5	0.5	9.6	18.8	0.3	(Hou et al., 2015), (Cao et al., 2019), (Ma et al., 2012), (Ma et al., 2010), (He et al., 2022), (Wei et al., 2018) , In this study
Permeable with coverage	15	1	10	1	9.6	18.8	0.3	
Impermeable without coverage	25	0.5	5	0.5	0	0	0	
mpermeable with coverage	15	1	10	1	0	0	0	
Manure treamtment								
Anaerobic digestion	5.1	0.5	5	0.5	0	0	0	(Cao et al., 2019), In this study
Composting	21.1	1	4.9	1	0	0	0	
Manure application								
Raw manure	25	1	15	0.21	9.6	18.8	0.3	(Hou et al., 2015), (Ma et al., 2012), (Ma et al., 2010), (Cao et al., 2019), (Hou et al., 2017), In this study
Anaerobic digestate manure	15	0.48	15	0.10	9.6	18.8	0.3	
Composting manure	1	1	15	0.21	9.6	18.8	0.3	

Table S18 Meta-results of the effect of different crude protein reductions on performance and egg quality in laying hens.

Indicators	CP reduction	Mean	SE	p.value	95%CI_Min	95%CI_Max
Egg Production	1%	0.03	0.033	0.343	-0.033	0.095
Egg Weight	1%	-0.01	0.009	0.484	-0.025	0.012
Egg Mass	1%	0.01	0.056	0.835	-0.100	0.123
Shell Strength	1%	0.03	0.021	0.138	-0.011	0.075
Feed Intake	1%	-0.01	0.018	0.778	-0.041	0.031
Feed Efficiency	1%	0.00	0.039	0.980	-0.077	0.079
Egg Production	1-2%	-0.02	0.019	0.194	-0.062	0.013
Egg Weight	1-2%	-0.02	0.005	0.002	-0.026	-0.006
Egg Mass	1-2%	-0.02	0.035	0.626	-0.088	0.053
Shell Strength	1-2%	0.01	0.012	0.353	-0.013	0.036
Feed Intake	1-2%	0.01	0.012	0.491	-0.016	0.033
Feed Efficiency	1-2%	-0.02	0.018	0.169	-0.060	0.011
Egg Weight	>2%	-0.05	0.005	0.000	-0.057	-0.038
Egg Production	>2%	-0.08	0.018	0.000	-0.118	-0.047
Egg Mass	>2%	-0.06	0.029	0.043	-0.117	-0.002
Shell Strength	>2%	-0.01	0.015	0.501	-0.040	0.020
Feed Intake	>2%	-0.07	0.011	0.000	-0.096	-0.051
Feed Efficiency	>2%	-0.08	0.016	0.000	-0.110	-0.049

Data sources and calculations of Nr losses

Data sources

Data related to domestic feed crop production (i.e., crop yield and agricultural inputs) were obtained from Chinese official statistics. Literature data were used for the emission parameters of agricultural inputs production when available; otherwise, values from the Ecoinvent 3 database were used. Nr losses factors during feed crop production and manure management were derived from Previous experiments of the research group (Table S10-S11). Feed formulas (Table S3) and manure management data (i.e., housing and storage types, and the adaption of treatment techniques, Table S6) were obtained from a survey conducted 91 farms and the experiment of this study.

Calculation

Quantification of feed crop production

$$NE_{crop,plant} = NE_{AI} + NE_{energy,con} + NE_{feed,crop} \quad \text{(S1)}$$

Where, $NE_{crop,plant}$, represents active nitrogen (g Nr) during feed production; NE_{AI}, respectively represents active nitrogen (g Nr) during the production of agricultural products; $NE_{energy,con}$ represents active nitrogen (g Nr) in the process of energy production; $NE_{feed,crop}$ represents active nitrogen (g Nr) during feed planting, respectively.

$$NE_{energy} = \sum x.z\, S_{crop,x} * EF_{N,energy,con,z} * W_{energy,con,k} \quad \text{(S2)}$$

$$S_{crop,x} = \frac{Q_x * Yield_x}{Ratio_x} \quad \text{(S3)}$$

Where x represents the type of feed crops (i.e. corn, soybean and wheat), y represents the types of agricultural products (e.g. chemical fertilizers, seeds and pesticides), z represents the type of energy input during the planting of feed crops (e.g. diesel, electric power, etc., Table S14). $S_{crop,x}$ represents the planting area of feed crop x (ha). $EF_{N,AI,y}$ y represents active nitrogen emission factor (g Nr kg per product^{-1}) (Table S7-S8). $EF_{N,energy,con,z}$ represents active nitrogen emission factor (g Nr unit energy^{-1}) in the production process of energy z respectively (Table S7). $W_{AI,z}$ and $W_{energy,con,k}$ represent the input of agricultural products y (kg ha^{-1}) and energy z (kg ha^{-1} or kwh ha^{-1} or MJ ha^{-1}) during the planting of feed crops (Table S11-13). Q_x represents feed consumption (kg); $Yield_x$ represents the yield of crop x (kg ha^{-1}) (Table S13). $Ratio_x$ represents the yield (%) of feed x.

$$NE_{feed,crop} = NE_{crop,NH_3} + NE_{crop,N_2O} + NE_{crop,NO_x} + N_{crop,erosion} + N_{crop,leaching} + NE_{crop,runoff} \quad (S4)$$

$$NE_{crop,NH_3} = \sum x \left(N_{fertilizer,x} * EF_{crop,NH_3}\right) * S_{crop,x} * Allocation_{eco,x} \quad (S5)$$

$$NE_{crop,dir,N_2O} = \sum x \left(N_{fertilizer,x} * EF_{crop,dir,N_2O}\right) * S_{crop,x} * Allocation_{eco,x} \quad (S6)$$

$$NE_{crop,NOx} = \sum x \left(N_{fertilizer,x} * EF_{crop,NOx}\right) * S_{crop,x} * Allocation_{eco,x} \quad (S7)$$

$$N_{input,x} = N_{fertilizer,x} + N_{BNF} + N_{deposition} + N_{irrigation} + N(P)_{seed,x} + N(P)_{straw,x} \quad (S8)$$

$$N(P)_{crop,leaching} = N(P)_{input,x} - N(P)O_{grain,straw,x} - NE_{crop,NH_3} - NE_{crop,dir,N_2O} - NE_{crop,NOx} \quad (S9)$$

$$NE_{crop,erosion} = \sum x \left(N_{input,x} - NO_{grain,straw,x}\right) * EF_{crop,N,erosion} * S_{crop,x} * Allocation_{eco,x} \quad (S10)$$

$$N_{crop,leaching} = N_{input,x} - NO_{grain,straw,x} - N_{crop,leaching} - NE_{crop,NH_3} - NE_{crop,dir,N_2O} - NE_{crop,NOx} \quad (S11)$$

$$NE_{crop,runnoff} = \sum x \begin{pmatrix} N_{input,x} - NO_{grain,straw,x} \\ -N(P)E_{crop,erosion,x} \end{pmatrix} * EF_{crop,N,runoff} * S_{crop,x} * Allocation_{eco,x} \quad \text{(S12)}$$

$$NE_{crop,leaching} = N_{input,x} - NO_{grain,straw,x} - NE_{crop,erosion,x} - NE_{crop,runoff,x} - NE_{crop,NH_3} - NE_{crop,dir,N_2O} - NE_{crop,NOx} \quad \text{(S13)}$$

$$NE_{crop,leaching} = \sum x\, N_{crop,leaching} * EF_{crop,N,leaching} * S_{crop,x} * Allocation_{eco,x} \quad \text{(S14)}$$

Where, NE_{crop,NH_3}, NE_{crop,dir,N_2O}, $NE_{crop,NOx}$ $NE_{crop,erosion}$, $N_{crop,leaching}$ and $NE_{crop,runoff}$ represent the loss of ammonia, nitrous oxide, nitrogen oxide, erosion, leaching and runoff caused by nitrogen inputs in the field during the planting of feed crops. $N_{fertilizer}$ is the N input by fertilizer (kg N ha^{-1}). $N_{input,x}$ represents the input of total nitrogen (kg N ha^{-1}) in the planting process of crop x. N_{BNF} is the N input by biological nitrogen fixation (kg N ha^{-1}). $N_{deposition}$ is the N input by atmospheric deposition. $N_{irrigation}$, $N_{seed,x}$, $N_{straw,x}$ are the N inputs by irrigation (kg N ha^{-1}), seed (kg N ha^{-1}), and recycled straw into the field (kg N ha^{-1}). $NE_{crop,erosion,x}$, $NE_{crop,runnoff}$ and $NE_{crop,leaching}$ represent the erosion, runoff and leaching nitrogen (kg N ha^{-1}) caused by the input of nitrogen during the planting. $NO_{grain,straw,x}$ represents the nutrient content of nitrogen (kg N ha^{-1}) output in the form of grain and straw (Table S9). EF_{crop,dir,N_2O} represents the emission factor (%) of direct emission of nitrous oxide caused by fertilizer application (Table S17). EF_{crop,NH_3} and NE_{crop,N_2O} represent the emission factors (%) of ammonia and nitrous oxide emissions caused by nitrogen input from farmland (Table S7). $EF_{crop,N,erosion}$, $EF_{crop,N,runoff}$ and $EF_{crop,N,leaching}$ represent the nitrogen

emission factors (%) that are lost by erosion, runoff and leaching due to the input of nitrogen (Table S16) $Allocation_{eco,x}$ represents the economic distribution coefficient of feed component x (for example, the distribution coefficient of soybean meal is 56%). Economic allocation is common allocation method used in the environmental life cycle evaluation of livestock products. Therefore, we used the economic allocation method in our study. Two allocations were performed in this study. The first allocation was used for wheat production only to divide the environmental impacts associated with crop cultivation into wheat pellets and straw. The second allocation was used for wheat and soybeans to separate the ecological effects of crop cultivation, processing, and land use into feed components and corresponding by-products. The allocation factors and mass ratios for the different products are shown in the supporting information (Table S5).

Quantification of feed processing and transportation

$$NE_{feed,processing,x} = \sum x, z \, EF_{energ,con,z} * W_{feed,processing,energ,z} * W_x * Allocation_{eco,x} \quad (S19)$$

$$NE_{feed,trans} = NE_{feed,truck} + NE_{feed,ship} \quad (S20)$$

$$NE_{feed,truck} = \sum x \, EF_{truck,NH3,x} * DT_{truck,x} * W_{feed,truck,x} * W_x * Allocation_{eco,x} \quad (S21)$$

$$NE_{feed,ship} = \sum x \, EF_{ship,NH3,x} * DT_{ship,x} * W_{feed,ship,x} * W_x * Allocation_{eco,x} \quad (S22)$$

Where, $NE_{feed,processing}$ represent the active nitrogen (g Nr) of the feed required by layer during processing. $W_{feed,processing,energ,z}$ represents the

energy consumption z during feed processing (kg kg^{-1} feed^{-1}. kwh kg^{-1}. MJ kg^{-1} feed^{-1}). W_x represents feed crop consumption (kg). $NE_{feed,trans}$ represent active nitrogen (g Nr) of the feed required by each layer during transportation. $EF_{truck,NH3}$ and $EF_{ship,NH3}$ represent the emission factor (%) of NH_3 during light truck and ship transportation (Table S8). $DT_{truck,x}$ and $DT_{ship,x}$ represent the total distance (km) of feed crops x transported by light trucks and ships, respectively. $W_{feed,truck,x}$ and $W_{feed,ship,x}$ represent the total weight (kg) of feed crops x transported by light trucks and ships, respectively.

Quantification of animal excretion

$$VS_{manure} = VS_{feed} * (1 - OMD\% + UE\%) \qquad (S23)$$

$$OMD\% = DMD\% + 3.97\% \qquad (S24)$$

Where, VS_{manure} (kg VS) indicates volatile solids (VS) excretion, VS_{feed} (kg VS) indicates feed VS intake, $OMD\%$ indicates feed VS digestibility (the percentage of feed VS intake that is not digested will be excreted as feces), and $UE\%$ indicates the urinary energy expressed as % of gross energy intake, assumed as 4% for layers (IPCC, 2006). $OMD\%$ was quantified based on its relationship with feed dry matter digestibility- $DMD\%$, based on the linear regressions determined from experimental studies of feed digestibility (IPCC, 2006). The $DMD\%$ for layers is 78.97% in China (IPCC, 2006). The amount of total carbon (TC) in animal excreta (VS_{manure}) per animal category was estimated according to the constant ratios of TC/VS ($f_{TC/VS}$) in excretion 0.58 for

layers (Sommer et al., 2013).

$$N_{manure} = N_{input,feed} - N_{egg} - N_{weight,gain} \quad (S25)$$

$$\mathrm{N_{egg}} = EGG * NC_{egg} \quad (S26)$$

$$N_{weight,gain} = LWG * NC_{meat} \quad (S27)$$

Where, N_{manure}(kg N) is the manure nutrient (nitrogen (N)) output. $\mathrm{NI_{feed}}$ (kg N) means feed nutrient intake. $N_{weight\ gain}$(kg N) is the nutrient retention in the live weight gain. Egg (kg) means producing egg. NC_{egg} (%) is the nutrient content in egg (Table S16). LWG (kg) means the live weight gain. NC_{meat} (%) is the nutrient content in live weight retention of animal (Table S15).

Quantification of laying hens and manure management

The environmental losses in the stage of laying hens breeding and feces and urine management come from three processes: feces and urine storage in the house, feces and urine storage and treatment outside the house, and feces and urine application. The calculation formula is as follows:

$$NE_{rearing} = NE_{house,energy} + NE_{manure,id} + NE_{manure,od} + NE_{application} + NE_{discharge} \quad (S28)$$

Where, $NE_{house,energy}$ represent active nitrogen (g Nr) loss caused by energy consumption in the house. $NE_{manure,id}$ represents the active nitrogen (g Nr) during storage in the feces and urine house. $NE_{manure,od}$ represents active nitrogen (g Nr) during the storage and treatment of feces and urine outside the house. $NE_{application}$ respectively represents active nitrogen (g Nr) in the process of returning manure and urine to the field. $NE_{discharge}$ represents the

active nitrogen (g Nr) produced by direct excretion of feces and urine.

$$NE_{manure,id} = NE_{manure,id,NH_3} + NE_{manure,id,leaching} \\ +NE_{manure,id,runoff} + +NE_{manure,id,erosion} \quad (S29)$$

$$NE_{manure,od} = NE_{treat} + NE_{stroage} \quad (S30)$$

$$NE_{treat} = NE_{AD,NH_3,N20,NOX} + NE_{com,NH3,N2O,NOX} \quad (S31)$$

$$NE_{AD,NH_3,N2O,NOX} = \left(N_{manure} - NE_{discharge} - NE_{manure,id}\right) * f_{AD} \\ * NE_{AD,NH_3,N2O,NOX} \quad (S32)$$

$$NE_{com,NH3,N2O,NOX} = \left(N_{manure} - NE_{discharge} - NE_{manure,id}\right) * f_{com} \\ * NE_{com,NH3,N2O,NOX} \quad (S33)$$

$$NE_{AD,indir\ N_2O} = \left(NE_{AD,slurry,NH_3} + NE_{AD,slurry,NO_X}\right) * EF_{GAS} \quad (S34)$$

$$NE_{com,indir\ N_2O} = \left(NE_{com,solid,NH_3} + NE_{com,solid,NO_X}\right) * EF_{GAS} \quad (S35)$$

$$NE_{stroage} = NE_{stroage,NH_3} + NE_{stroage,N_2O} + NE_{stroage,NO_X} \\ +NE_{stroage,leaching} + NE_{stroage,runnoff} + NE_{stroage,erosion} \quad (S36)$$

$$NE_{application} = NE_{app,NH_3} + NE_{app,N_2O} + NE_{app,NO_X} \\ +NE_{app,leaching} + +NE_{app,runoff} + NE_{app,eronsion} \quad (S37)$$

In the formula, N_{manure} represents the total excretion of nitrogen in the feces and urine, which is calculated according to the difference between the total input of nitrogen and phosphorus in the feed and the nitrogen and phosphorus content of the layer body. $NF_{manure,id,NH3}$ represents the loss of NH3 (g N-NH_3) caused by the storage of feces and urine. $NF_{manure,id,\ leaching}$, $NF_{manure,id,\ runoff}$ and $NF_{manure,id,\ eronsion}$ represent the leaching, runoff and erosion loss of nitrogen (g Nr) caused by the storage in the manure and urine house. NF_{treat} and $NF_{stroage}$ represent the loss of nitrogen (g Nr) during the treatment and storage of feces and urine, respectively. $N_{AD,NH3,N2O,NOX}$ and $NF_{com,NH3,N2O,NOx}$ respectively represent the emissions of NH_3 (g N-NH_3), N_2O (g N-N_2O) and NO_X (g N-NO_x) in the process of feces biogas fermentation and feces composting. f_{AD} and f_{com} respectively

represent the proportion of biogas fermentation treatment and the proportion of composting treatment (%). $EF_{AD,NH3,N2O,NOX}$, $EF_{AD,NH3,N2O,NOX}$ and $EF_{com, NH3,N2O,NOX}$ respectively represent N-NH_3, N-N_2O, N-NO_X emission factors (%) in biogas fermentation process and fecal composting process(Table S17). $NF_{storage, runoff}$, $NF_{storage, leach}$ and $NF_{storage, erosion}$ represents the loss of NH_3 (g N-NH_3), N_2O g N-N_2O and NOx g N-NOx) during the storage of feces and urine. $NF_{storage, runoff}$, $NF_{storage, leaching}$ and $NF_{storage, erosion}$ represents the leaching, runoff and erosion loss of nitrogen (g Nr) during the storage of feces and urine outside the house. $NF_{app, NH3}$, $NF_{app, N2O}$ and $NF_{app, NOX}$ represents the loss of NH_3 (g N-NH_3), N_2O (g N-N_2O)and NO_X (g N-NO_x) in the process of returning manure and urine to the field. $NF_{app, leaching}$, $NF_{app, runoff}$ and $NF_{app, eronsion}$ represents the leaching, runoff and erosion loss of nitrogen (g Nr) in the process of returning manure and urine to the field.

References

Cappelaere, L., Le, J., Grandmaison, C., Martin, N., Lambert, W., 2021. Amino Acid Supplementation to Reduce Environmental Impacts of Broiler and Pig Production: A Review. 8, pp. 1-14.

Chen, S.; Lu, F.; Wang, X. K., 2015. Estimation of greenhouse gases emission factors for China's nitrogen, phosphate, and potash fertilizers. Acta Ecologica Sinica, 35, (19), 6371-6383 (in Chinese with English abstract).

China animal husbandry and veterinary yearbook (in Chinese);In Ministry of

Agriculture and Rural Affairs of the People's Republic of China: Beijing, China, 2018.

China energy statistical yearbook, 2016. In China Statistics Press: Beijing, China (in Chinese).

Guidelines for the Life Cycle Inventory of Agricultural Products. Version 2.0, July 2014.

Gustavsson, J., Cederberg, C., Sonesson, U., Van Otterdijk, R., Meybeck, A., 2011. Global food losses and food waste.

Huang, X., Song, Y ., Li, M., Li, J., Hou, Q., Cai, X., Zhu, T., Hu, M., Zhang, H., 2012. A high-resolution ammonia emission inventory in China. Global Biogeochemical Cycle. 26, 239-256.

IPCC, 2006. IPCC Guidelines for National Greenhouse Gas Inventories, Volume 4Agriculture, Forestry and Other Land Use. IGES, Kanagawa.

Jiang, J. H., 2016. Spatio-temporal models for estimating footprint of nitrous oxides. Ph.D. thesis, Zhejiang University, Hangzhou, Zhejiang (in Chinese with English abstract).

Li, Q., 2019. Preliminary study on root-crown ratio of main green manure. Forum of South China, 50(13):23+32 (in Chinese).

Li, X. Y.; Li, H. P., 2012. Temporal and spatial distribution characteristics of ammonia and nitrous oxide emissions in China's atmospher. China Environmental Science, 32, (1), 37-42 (in Chinese with English abstract).

Liang, L., 2009. Environmental impact assessment of circular agriculture based

on life cycle assessment: methods and case studies. China Agricultural University (in Chinese with English abstract).

Liu, X., Sun H., Zhang, X., Zhang L., 2013. Effects of water and nitrogen on root/shoot ratio and water use efficiency of winter wheat. Chinese Journal of Eco-Agriculture, 21(3): 282-289 (in Chinese with English abstract).

Ma, Z. H., 2002. Comparison of GHG emission factor of the main energy in China. China Atomic Energy Science Academy (in Chinese with English abstract).

National Development and Reform Commission (NDRC), 2018. Compilation cost and benefit data of National agricultural product. China Statistics Press (in Chinese).

Nemecek, T., Bengoa, X, Lansche, J, Mouron, P, Rossi, V., Humbert, S., 2014. Methodological

Nemecek, T.; Kägi, T., 2007. Life Cycle Inventories of Swiss and European Agricultural Production Systems; Final Report Ecoinvent Report v2.0, no.15; Agrosope Reckenholz-Tänikon Research Station ART: Zürich and Dübendorf, Switzerland.

Nemecek, T.; Kägi, T., 2007. Life Cycle Inventories of Swiss and European Agricultural Production Systems; Final report Ecoinvent report v2.0, no.15; Agrosope Reckenholz-Tänikon Research Station ART: Zürich and Dübendorf, Switzerland.

Sommer, S.G., Christensen, M.L., Schmidt, T., Jensen, L.S., 2013. Animal

Manure Recycling: Treatment and Management. 382 pp.

Thomas, N.; Jens, L.; Patrik, M.; Eliane, R.,2015. World Food LCA Database: Methodological Guidelines for the Life Cycle Inventory of Agricultural Products.

Trabue, S.L., Kerr, B.J., Scoggin, K.D., Andersen, D., van Weelden, M., 2021. Swine diets impact manure characteristics and gas emissions: part I protein level. Sci. Total Environ. 755, 142528.

Van Harn, J., Dijkslag, M.A., Van Krimpen, M.M., 2019. Effect of low protein diets supplemented with free amino acids on growth performance, slaughter yield, litter quality, and footpad lesions of male broilers. Poult. Sci. 98, 4868–4877.

Wang J., 2019. Research on optimization of land use structure in Quzhou County based on carbon balance. China Agricultural University (in Chinese with English abstract).

Wang, Q., Shao, D., Tong H B., Zhang, A., 2017. Effects of different dung cleaning mode on envirnment quality of chicken house and components of chicken dung. Guizhou Agricultural Sciences, 45(1), 87-90 (in Chinese with English abstract).

Wang, X., Ndegwa, P.M., Joo, H.S., Neerackal, G.M., Harrison, J.H., Stöckle, C.O., Liu, H., 2016. Reliable low-cost devices for monitoring ammonia concentrations and emissions in naturally ventilated dairy barns. Environ. Pollut. 208, 571–579.

Wang, Z. B.; Chen, M.; Chen, F., 2015. Carbon Footprint Analysis of Crop Production in North China Plain. Scientia Agricultura Sinica, 48, (1), 83-92 (in Chinese with English abstract).

World Food LCA Database (WFLDB). Quantis and Agroscope, Lausanne and Zurich,

Yang, C. Y.; Lin, X.; Dong, Z. F.; Wang, J. F., 2018. Research on characteristics and division analysis of provincial nitrous oxide emissions in electricity industry China. Ecology and Environmental Science, 27, (9), 1688-814 1697 (in Chinese with English abstract).

Zhou, Y., 2018. Carbon Footprint Assessment Method and Case study of Intensive Pig Production System in China. Chinese Academy of Agricultural Sciences (in Chinese with English abstract).
